# Dr. John Homans

**Published:** 1902-06

**Authors:** 


Dr. John Homans, 2d, of Boston, died in that city May 4, 1902, aged 45 years. Dr. Homans was a physician of prominence, held many important professional and institutional positions, was a member of the Society of the Cincinnati, and was greatly respected by his colleagues and other friends.

